# Improving B-cell epitope prediction and its application to global antibody-antigen docking

**DOI:** 10.1093/bioinformatics/btu190

**Published:** 2014-04-21

**Authors:** Konrad Krawczyk, Xiaofeng Liu, Terry Baker, Jiye Shi, Charlotte M. Deane

**Affiliations:** ^1^Department of Statistics, Oxford University, OX1 3TG, Oxford, ^2^UCB Pharma, SL1 3WE Slough, UK and ^3^Shanghai Institute of Applied Physics, Chinese Academy of Sciences, Shanghai 201800, China

## Abstract

**Motivation:** Antibodies are currently the most important class of biopharmaceuticals. Development of such antibody-based drugs depends on costly and time-consuming screening campaigns. Computational techniques such as antibody–antigen docking hold the potential to facilitate the screening process by rapidly providing a list of initial poses that approximate the native complex.

**Results:** We have developed a new method to identify the epitope region on the antigen, given the structures of the antibody and the antigen—EpiPred. The method combines conformational matching of the antibody–antigen structures and a specific antibody–antigen score. We have tested the method on both a large non-redundant set of antibody–antigen complexes and on homology models of the antibodies and/or the unbound antigen structure. On a non-redundant test set, our epitope prediction method achieves 44% recall at 14% precision against 23% recall at 14% precision for a background random distribution. We use our epitope predictions to rescore the global docking results of two rigid-body docking algorithms: ZDOCK and ClusPro. In both cases including our epitope, prediction increases the number of near-native poses found among the top decoys.

**Availability and implementation:** Our software is available from http://www.stats.ox.ac.uk/research/proteins/resources.

**Contact:** deane@stats.ox.ac.uk

**Supplementary information:** Supplementary data are available at *Bioinformatics* online.

## 1 INTRODUCTION

Antibodies are the key protein factors in the acquired immune responses in vertebrates. The most common human antibody isotype is the IgG, which is one of the main mediators of secondary immune responses (Kuroda *et al.*, 2012; Raghunathan *et al.*, 2012; Sela-Culang *et al.*, 2013). Antibodies have a conserved structure with >1700 solved structures available in the Protein Data Bank (Berman *et al.*, 2000; Dunbar *et al.*, 2013). Most of the variability in antibodies (both sequence and structure) can be found in the antigen binding site, which is chiefly composed of the complementarity-determining region loops (CDRs) (Raghunathan *et al.*, 2012). The affinity and specificity of the antibody’s cognate antigen can be effectively modulated by only a few mutations to the CDRs (Raghunathan *et al.*, 2012). Owing to their malleable binding properties, antibodies are currently one of the most important biopharmaceuticals (Murad *et al.*, 2012; Wark and Hudson, 2003).

The majority of the technologies used for artificial antibody design are based on costly screening campaigns. However, there is a growing number of computational methods aimed at aiding the process of artificial antibody design (Kuroda *et al.*, 2012). Two areas of computational antibody design are the focus of this manuscript: B-cell epitope prediction (e.g. EL-Manzalawy and Honavar, 2010; Yao *et al.*, 2013) and global antibody–antigen docking (e.g. Brenke *et al.*, 2012).

Given a sequence or structure of an antigen, *in silico* B-cell epitope prediction aims to identify a set of residues on the antigen capable of binding an antibody (Kringelum *et al.*, 2012). Many successful B-cell epitope prediction methods rely on structural information but sequence alone can also produce useful predictions (Lin *et al.*, 2013). The majority of current methods operate without antibody information, aiming to identify all potential antibody binding sites (Kuroda *et al.*, 2012; Sela-Culang *et al.*, 2013). Attempting to map all epitopes might not be optimal because some antigens, such as hen egg white lysozyme, have been shown to form complexes with many different antibodies. These bind to different areas, meaning that most of the lysozyme’s surface constitutes a part of some epitope (Sela-Culang *et al.*, 2013). Moreover, it has been shown that two different therapeutic antibodies, Gevokizumab and Canakinumab, activate two distinct pathways by binding to different epitopes of IL-1β (Blech *et al.*, 2012). In this article, we create antibody-specific epitope predictions, as we believe these will be more useful for the development of therapeutic antibodies (Soga *et al.*, 2010; Zhao and Li, 2010; Zhao *et al.*, 2011).

Computational B-cell epitope prediction provides information about the regions of the antigen bound by the antibody but it does not directly contribute to the knowledge of the particular antibody residues that need to be mutated so as to modify its function. This problem can be tackled by antibody–antigen docking, which, given the structure of the antibody and the antigen, provides a list of putative orientations of the two molecules with respect to each other. Antibody–antigen docking requires different methodology from that used for the corresponding problem concerning non-antibody targets (Brenke *et al.*, 2012; Mendez *et al.*, 2005). This is because antibodies use different residues in their binding sites when compared with both general proteins and antigens and thus an asymmetric scoring system is required that accounts for these discrepancies (Brenke *et al.*, 2012; Krawczyk *et al.*, 2013).

In this manuscript, we focus on epitope prediction and global docking and how those two methods in concert can facilitate computational artificial antibody design. We develop an antibody-specific epitope prediction method EpiPred, which uses geometric matching of the antibody and antigen interfaces coupled with an antibody–antigen-specific knowledge-based potential. We use our epitope predictions to rescore the global docking results of two fast rigid-body docking algorithms, ZDOCK and ClusPro server in antibody mode (Brenke *et al.*, 2012; Chen *et al.*, 2003). We demonstrate that including the epitope information in our global docking pipeline enriches the top decoys with more native poses.

## 2 METHODS

### 2.1 Data

A non-redundant dataset of crystal structures was downloaded from the structural antibody database (SAbDAb; Dunbar *et al.*, 2013) in August 2013. The complexes were selected such that no two antibodies shared >99% sequence identity and the corresponding antigens shared not >90% sequence identity. All antigens were proteins as defined by SAbDab (>50 residues) and the complexes had to be of resolution 3 Å or better. The final dataset consisted of 148 structures (SAbDab-nr), 30 ofwhich were chosen at random to constitute the test set, referred to as X-test. The homology model dataset, H-test, consisted of 15 antibody–antigen complexes as used by Krawczyk *et al.* (2013) and Sircar and Gray (2010). These are model structures built with RosettaAntibody (Sivasubramanian *et al.*, 2009). The homology models we obtained did not have the H3 loop modeled so these were modeled using FREAD (Choi and Deane, 2011, 2010). The PDB codes and the corresponding chains of structures used in this study are given in the Supplementary Section 1.

### 2.2 Epitope prediction

#### 2.2.1 Epitope prediction algorithm

Our epitope prediction algorithm is a combination of geometric fitting and a knowledge-based asymmetric antibody–antigen scoring. The algorithm is divided into three steps.

**Fig. 1.**
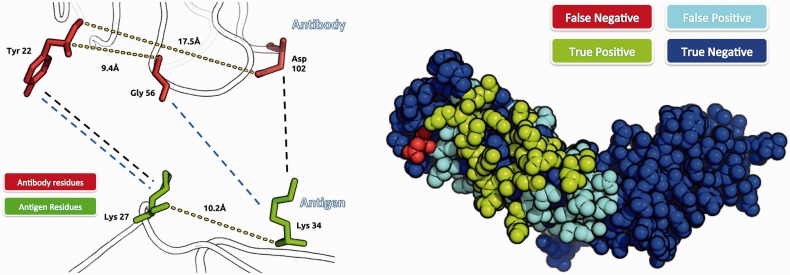
Left: Example of a case when intramolecular distances can provide information about which intermolecular contacts can exist. The antibody–antigen contacts between Tyr-22 and Lys-27 and Gly-56 and Lys-34 (blue dashes) can exist as the intramolecular distance between Tyr-22 and Gly-56 is 9.4 Å and the distance between the two Lys residues is 10.2 Å. The difference between those two intramolecular distances is 0.8 Å, which is below the cutoff of 1 Å. As a counterexample, the contacts between Tyr-22 and Lys-27 and Asp-102 and Lys-34 (black dashes) cannot be satisfied simultaneously because the intramolecular distance between Tyr-22 and Asp-102 is 17.5 Å. Right: The top epitope prediction for the antigen 1boy (human tissue factor, the unbound form of the antigen complexed in 1ahw in H-test). The prediction consists of a set of residues, which are considered to constitute the general area of the epitope. The true positives are shown in green, false positives in teal, false negatives in red and true negatives in dark blue. This prediction achieved 36% precision and 94% recall. (The target comes from the dataset H-test, and thus, the antibody used in the prediction was a homology model and the corresponding antigen was in the unbound form)

Firstly, epitope-like surface patches on the antigen are enumerated. These are designed to be roughly the same size as the approximate epitopes used in our earlier local docking study (Krawczyk *et al.*, 2013). An epitope is defined here as surface antigen residues (>7% Δ ASA) whose heavy atoms are within 4.5 Å of a heavy atom on the antibody. These candidate epitope patches are then scored using geometric fitting and a specific antibody–antigen score. The geometric fit is calculated by enumerating all possible contacts between the set of putative epitope residues and the CDRs and evaluating which pairs of antibody–antigen contacts can be satisfied simultaneously (see Fig. 1 for an example). The final epitope score for each patch is a sum of all possible contacts between the given epitope and CDRs, weighted by the number of other contacts they can satisfy simultaneously as well as the antibody–antigen Precision Score for the particular amino acid contact pair. The Precision Score has been adapted from our earlier work on local antibody–antigen docking (Krawczyk *et al.*, 2013). In the final ranking of the candidate epitopes, we only keep patches with <30% of their residues in common.

#### 2.2.2 Enumerating epitope-like patches

Our algorithm identifies plausible extended epitope-like patches on the surface of the antigen and annotates each of them with a score indicating the likelihood of containing the correct epitope. A putative epitope patch is created for every surface residue on the antigen. The patch consists of the neighborhood of the surface residues and is constructed by selecting every surface residue within 4.5 Å of the chosen residue. This step is followed by adding the surface residues within 4.5 Å of the current residues in the patch. See Supplementary Section 2 for details.

#### 2.2.3 Precision Score for the epitope prediction

EpiPred uses geometric matching of the antibody and antigen surfaces, which are weighted by a specific antibody–antigen score. The antibody-specific score is the Precision Score, which we have previously shown to be able to more reliably identify antibody-specific docking poses (Krawczyk *et al.*, 2013). Here 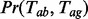 denotes the likelihood of the docking algorithm to correctly pair a residue of type *T_ab_* on the antibody and a residue of type *T_ag_* on the antigen (for instance, glycine on the antibody and serine on the antigen). The Precision Score 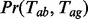 was estimated by executing ZDOCK on each of the 118 targets in SAbDab-nr that were not in X-test and in the set of top 200 ZDOCK-scored poses counting how many times a given pair of residues was matched correctly with respect to the native structures. For details of the procedure see Supplementary Section 3.

To ensure we have not overtrained the Precision Score for the H-test dataset, we have removed all members of the SAbDab-nr that had >90% sequence identity with any antigen and >99% with any antibody in the H-test. The sequence identity was calculated using CD-HIT (Li and Godzik, 2006).

#### 2.2.4 Scoring putative epitopes

Let *Epi* denote the set of residues in a putative epitope and *Ab* the set of residues supplied as the binding site on the antibody. We create a graph *G* where each node *n*, corresponds to an element of the Cartesian product of *Epi* and *Ab*: 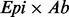. Thus, if there was a tyrosine (Y) residue in *Epi* and a histidine (H) residue in *Ab*, there will be a node *n′* in *G* that corresponds to this pair—(*Y*,*H*). Each of the nodes represents a possible intermolecular contact between antibody and antigen residues.

We add an edge between any two nodes in *G* if the antibody–antigen contacts defined by those nodes can be geometrically satisfied at the same time. Take node *n*_1_, which stands for a contact between antibody residue *r_ab_*_1_ and antigen residue *r_ag_*_1_ and node *n*_2_ with antibody residue *r_ab_*_2_ and antigen residue *r_ag_*_2_. Define 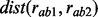 as the intramolecular distance between the two residues *r_ab_*_1_ and *r_ab_*_2_. We place an edge between *n*_1_ and *n*_2_ only if the difference in intramolecular distances on the antibody and the antigen is below 1 Å as given by (1).

The choice of 1 Å was motivated by our analysis of the intramolecular distances between residues in contact. We concluded from this analysis that 1 Å offers a good balance between the coverage of the binding site and the number of residues that can satisfy this condition (Supplementary Section 4).
(1)
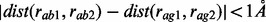



Let *d*(*n*) denote the degree of node *n*. The final score for a putative epitope *Epi* is given by (2).
(2)
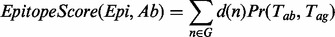

where *T_ab_* and *T_ag_* are the amino acid types of the antibody and antigen residues, respectively, which belong to node *n*.

The epitopes are ordered by their score and the top three non-overlapping epitopes are kept. Overlapping epitopes are defined as those that share >30% of the same residues with respect to the epitope with the higher epitope score.

We use our epitope prediction algorithm on each of the targets in X-test and H-test.

#### 2.2.5 DiscoTope 2.0 epitope predictions

The structures of 30 antigens in X-test were submitted to the DiscoTope 2.0 (http://www.cbs.dtu.dk/services/DiscoTope/; Kringelum *et al.*, 2012) server using the five thresholds suggested by the service: −3.7, −2.5, −1.0, 0.5 and 1.1. The residues predicted by DiscoTope as part of an epitope were compared with the native contacts (<4.5 Å from a heavy atom on the antibody). The best results, by Matthews correlation coefficient (MCC; Matthews, 1975), were obtained for the threshold of −3.7 and thus were used for the comparison with EpiPred.

#### 2.2.6 Evaluating the performance of antibody-specificity of EpiPred predictions

We have tested the ability of EpiPred to identify the epitopes of different antibodies on the same antigen using the test case of lysozyme. There are five standard (non-camelid) antibody–lysozyme complexes available in the PDB [we chose representatives: 1a2y, 1j1x, 1jhl, 1p2c and 2iff by clustering by sequence identity (99%) and removing any with missing binding site residues or high B-factors. We randomly select a structure from each cluster]. They bind to three distinct epitopes: epitope I (1a2y and 1jhl), epitope II (1p2c and 2iff) and epitope III (1j1x) (see Supplementary Section 5).

We retrained EpiPred using our original set of antibody-antigen complexes, removing any antibody with >99% sequence identity to the targets and all antigens with >90% sequence identity to lysozyme. We then ran EpiPred on each of the five cases.

### 2.3 Global docking

The global docking pipeline we have developed is divided into three steps. Firstly, up to three candidate epitope predictions from EpiPred are computed. Secondly, we perform global docking using a fast rigid-body algorithm (ZDOCK or ClusPro). We do not provide any epitope information at this point, only supplying the CDR residues to be masked. The final step consists of rescoring the poses produced by the docking algorithms using AB DockSorter.

The input to AB DockSorter consists of a single antibody–antigen pose supplied by the docking algorithm, a set of Chothia CDR residues and a set of residues for one of the predicted epitopes. For each pose, the AB DockSorter score is computed for each of the top three predicted epitopes, as given by EpiPred. The final score of a pose is the highest of the three scores. The poses for a given target are then reranked by this score.

#### 2.3.1 Docking algorithms

All the targets were subject to a random rotation and translation before submission to either ZDOCK or ClusPro. ZDOCK was run on all of the targets in the X-test set with the constraint on the antibody of the Chothia CDRs and with no epitope information. The software was executed using its default parameters, with the exception that the number of poses to probe was set to 10 000. As it was computationally feasible, we performed five runs of ZDOCK for each target, using different random seeds each time. An analogous procedure was applied to the targets in H-test. The targets in X-test and H-test were also submitted to ClusPro in antibody mode, using automatic CDR masking.

#### 2.3.2 Rescoring decoys

Consider a set of decoys *D* returned by either ZDOCK or ClusPro for a given target. We collect the top *N* decoys from *D* as ordered by the docking method. For a given decoy *d* in the set of top *N* decoys from *D*, let *Ab* denote the set of residues used as the antibody constraint and *Epi* a set of predicted epitope residues. Let 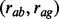 be any pair or residues in *d*, where 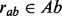 and 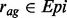. If the distance between *r_ab_* and *r_ag_* is observed to be <4.5 Å in the decoy *d*, this pair of residues contributes the value of 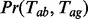 to the score for this decoy, where *T_ab_* is the type of the amino acid type of *r_ab_* and *T_ag_* is the amino acid type of *r_ag_*. If we let 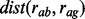 denote the distance in Ångstroms between residues *r_ab_* and *r_ag_*, the score for decoy *d* using antibody constraint *Ab* and epitope prediction *Epi* can be formalized by (3).
(3)
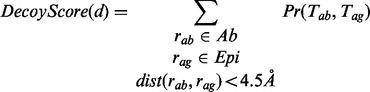



The top *N* decoys for a given target are given scores using our three epitope predictions. For each decoy, we retain the highest score of the three. We then use those scores to reorder the top *N* decoys for a given target.

In the case of ZDOCK for both X-test and H-test, we rescore the top 30 decoys for each target. We use the top 20 predictions for ClusPro, as this is the maximum number of decoys returned in most cases.

#### 2.3.3 Evaluation criteria for docking

To evaluate the quality of each decoy, we use the interfacial root mean square deviation (*I_rmsd_*), one of the metrics used in the Critical Assessment of PRotein Interactions (CAPRI) experiment (Mendez *et al.*, 2005). The value of *I_rmsd_* is the root mean square deviation between the interface region of the decoy and the native structure when those regions are optimally superimposed. The interface regions are defined as those within neighborhood of 10 Å from any residue on the binding partner.

We define a close-to-native decoy in the same way as the authors of the ClusPro antibody study (Brenke *et al.*, 2012). A close-to-native decoy is defined as having *I_rmsd_* <10 Å from the native complex. For each target in our test sets, we have the raw list of top *N* decoys as ordered by the docking algorithm and our rescored version thereof. To evaluate which ordering is better, we count the number of close-to-native decoys in the first, top five and top ten entries in the raw and rescored lists. For instance, if using our rescored list we find three close-to-native decoys in the top five and only one in the top five decoys in the raw list, we consider the rescoring to have improved the result. If in the top *N* of both lists no close-to-native decoy is found, we state there was no suitable decoy.

Because multiple runs were performed for ZDOCK, the reported results are derived from the comparison of the averages of close-to-native decoys in the raw and rescored lists for each target.

## 3 RESULTS

### 3.1 Epitope prediction

The epitope prediction algorithm presented here was inspired by our earlier work on local antibody–antigen docking, where we showed that it was possible to select close-to-native decoys when docking the antibody into an approximate region of the epitope (Krawczyk *et al.*, 2013).

To extend our local docking methodology to global docking, we have developed an epitope prediction algorithm that identifies surface patches on the antigen similar to the approximate epitopes used in our earlier work (see Fig. 1). Our epitope prediction algorithm receives, as input, the structure of an antibody and an antigen and returns a ranked list of epitope-like regions.

In our case, the aim is to generate epitope predictions specific for a given antibody to facilitate docking. Thus, the use of antibody information is crucial. However, as shown later, the antibody structure can be a homology model.

#### 3.1.1 Evaluation of the performance of epitope prediction

For our epitope prediction method to be applicable in virtual screening, it must be able to produce results given nothing more than the sequence of the antibody and the structure of the antigen. To verify this claim, we have evaluated the results of our epitope prediction algorithm on two datasets: a crystal structure dataset (X-test) and on homology model dataset (H-test). The first dataset (X-test) consisted of 30 non-redundant solved crystal structures of antibody–antigen complexes. The second dataset (H-test) consisted of 15 antibody–antigen targets, where the antibody is a RosettaAntibody (Sivasubramanian *et al.*, 2009) model with FREAD prediction for the H3 loop (Choi and Deane, 2011, 2010) and where 10 of the 15 antigens are in the unbound form.

The crystal structure dataset, X-test, constitutes the simpler of the two test sets, as all the structures are in their bound conformations. The results on the crystal structure dataset show the performance of the algorithm given close to perfect information for both structures and, as such, serve as a contrast to the homology dataset, H-test. The homology dataset poses the realistic challenge, as it represents the input that might be given to the algorithm in the course of virtual screening. The results from evaluating our epitope prediction algorithm on the crystal structure dataset are presented in Table 1.

**Table 1.** Table summarizing the results of epitope prediction on the X-test set

PDB	Ag size	Epitope prediction						Random	
		EpiPred			DiscoTope 2.0				
		Precision (%)	Recall (%)	MCC	Precision (%)	Recall (%)	MCC	Precision (%)	Recall (%)
4hj0	92	**32**	**90**	0.27	0	0	0.0	29	50
1tzh	94	1	6	0.04	**73**	**87**	0.72	12	25
4am0	96	**13**	**70**	0.09	33	20	0.19	14	60
2ih3	97	**16**	**64**	0.08	0	0	0.0	15	27
4i77	97	**23**	**55**	0.0	0	0	0.0	21	31
3q1s	113	**19**	**81**	0.15	0	0	0.0	20	37
1p2c	129	0	0	0.0	**100**	**5**	0.0	39	32
4ht1	131	**5**	**14**	0.05	0	0	0.0	28	44
3ab0	136	**33**	**73**	0.34	0	0	0.0	33	52
1v7m	145	**26**	**77**	0.29	0	0	0.0	9	16
4g3y	148	3	8	0.04	**100**	**17**	0.33	11	25
2vxt	156	4	9	0.04	**47**	**36**	0.3	14	23
3u9p	169	**31**	**100**	0.47	6	5	0.0	8	15
3o2d	178	**32**	**64**	0.28	0	0	0.0	9	16
1fns	196	0	0	0.0	**100**	**7**	0.0	33	11
3ma9	197	0	0	0.0	0	0	0.0	21	33
3rvv	223	**25**	**93**	0.39	15	17	0.07	6	15
3raj	230	0	0	0.0	0	0	0.0	24	21
1nfd	239	7	23	0.04	**92**	**70**	0.75	10	15
3i50	273	0	0	0.0	0	0	0.0	1	6
3gjf	276	**15**	**66**	0.2	5	11	0.05	6	15
3liz	329	**26**	**68**	0.34	0	0	0.0	10	15
3pgf	358	**2**	**4**	0.0	0	0	0.0	13	18
3zkm	375	**32**	**88**	0.46	0	0	0.0	11	15
3r1g	381	**37**	**100**	0.57	0	0	0.0	7	9
4jr9	409	19	85	0.34	**46**	**50**	0.46	4	11
4ene	442	0	0	0.0	0	0	0.0	1	5
3o0r	449	**8**	**70**	0.19	0	0	0.0	2	14
3t3p	453	0	0	0.0	**25**	**4**	0.0	4	6
1n8z	581	0	0	0.0	0	0	0.0	6	5

*Note:* We present the top EpiPred prediction and the corresponding results for DiscoTope 2.0 using a score threshold of −3.7. The values in bold indicate the best prediction result. Precision and recall were computed by the following formula: 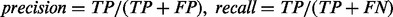 where TP stands for true positives, FP for false positives and FN for false negatives. In each case, we also give the Matthews correlation coefficient [MCC (Matthews, 1975)]. As control, the corresponding result using randomized score is given for each target.

As one would expect, it is easier to obtain good predictions on small antigens, as there are fewer candidate patches to enumerate. Similarly, one would expect a considerable drop of performance on the larger antigens because the method needs to distinguish between many more candidate patches. In cases such as 3t3p and 3pgf, which have 453 and 358 residues, respectively, no acceptable predictions were obtained. However, for 3liz, 3jr9, 3zkm and 3r1g, which have >300 residues, reasonable epitope predictions were generated, suggesting that the method retains a degree of predictive power even for larger antigens.

To give an indication of the background random distribution, we have executed EpiPred on each target in the crystal structure test set 500 times, randomizing the epitope score given to each candidate patch. For each run, we have averaged the precision and recall metrics of the top epitope of the 30 targets in X-test. The mean precision and recall averaged over 500 random-score runs on dataset X-test are 23 and 14%, respectively (the random results for each target are in Table 1). The corresponding average values for the first epitope prediction from Table 1 are 44% recall and 14% precision. The recall is considerably higher using our method, indicating its predictive power. Furthermore, the score correctly identifies better epitopes because the corresponding values for the second epitope are recall 25% and precision 13% and recall 18% and precision 7% for the third epitope (see Supplementary Section 6).

We have compared the results of EpiPred with one of the leading conformational B-cell predictors, DiscoTope 2.0 (Kringelum *et al.*, 2012). The results presented in Table 1 are the best prediction results from among the five thresholds proposed on the DiscoTope Web site (see Methods Section). On dataset X-test, EpiPred achieves better prediction results than DiscoTope 2.0 on 17 targets, worse on eight and neither of the methods produces a usable prediction on the remaining five. This indicates the value of using antibody structure information in epitope prediction.

In Supplementary Table S10, the results for epitope predictions for the homology cases (H-test) are given. The average precision and recall for the top predicted epitope are 16 and 47%, respectively. These results are similar (in fact not statistically significantly different; see Supplementary Section 7) to those achieved on the crystal structure set, indicating that the imprecise structural information from homology models and unbound antigens does not adversely affect the method.

#### 3.1.2 Specificity of EpiPred predictions

We have tested EpiPreds capacity to distinguish between the epitopes of different antibodies binding to the same antigen. As a test case we have picked lysozyme and the five distinct antibodies that bind to three different epitopes: epitope I (1a2y and 1jhl), epitope II (2iff and 1p2c) and epitope III with 1j1x (see Supplementary Section 5).

The results are shown in Table 2. Note that as the training set for these predictions is larger than that used for X-test (see Section 2), EpiPred produces a better prediction for 1p2c. EpiPred fails to predict epitope III and predicts a site that overlaps marginally with epitope I. In the case of epitopes I and II (which are on opposite sides of lysozyme), EpiPred correctly identifies the relevant part of the protein.

**Table 2.** Comparison of the specificity of EpiPred predictions evaluated on its capacity to distinguish between antibodies binding to lysozyme: epitope I (1a2y and 1jhl), epitope II (1p2c and 2iff) and epitope III (1j1x)

Epitope	Prediction from	Epitope I		Epitope II		Epitope III
Evaluated on	PDB	1a2y	1jhl	1p2c	2iff	1j1x
Epitope I	1a2y	**0.53**	–	0	0	0.19
	1jhl	–	**0.12**	0	0	0
Epitope II	1p2c	0.01	0.01	**0.15**	–	0
	2iff	0	0	–	**0.47**	0
Epitope III	1j1x	**0.17**	0.1	0	0	0

*Note:* The row indicates the antibody for which the EpiPred prediction was performed and the column the antibody with respect to which the prediction was evaluated using MCC.

### 3.2 Improving global docking using epitope predictions

We have used EpiPred to constrain the results of two fast rigid-body docking algorithms: ZDOCK and ClusPro (Brenke *et al.*, 2012; Chen *et al.*, 2003). ZDOCK is not optimized for docking antibody–antigen complexes beyond CDR masking, but as we have shown in our earlier work, its results can be rescored to enrich the top poses with close-to-native antibody–antigen complexes (Krawczyk *et al.*, 2013). ClusPro’s global antibody–antigen docking mode has been shown to be currently the best method in this area (Brenke *et al.*, 2012).

#### 3.2.1 Evaluating the performance of our global pipeline

We have evaluated the performance of our global docking pipeline based on the criteria introduced in the ClusPro study (Brenke *et al.*, 2012). We call an antibody–antigen pose close-to-native if its interfacial root mean square deviation (*I_rmsd_*) is <10 Å. We focus on the number of near-native poses found in the top *N* predictions, as these decoys could then be passed on for further refinement by flexible methods.

**Fig. 2.**
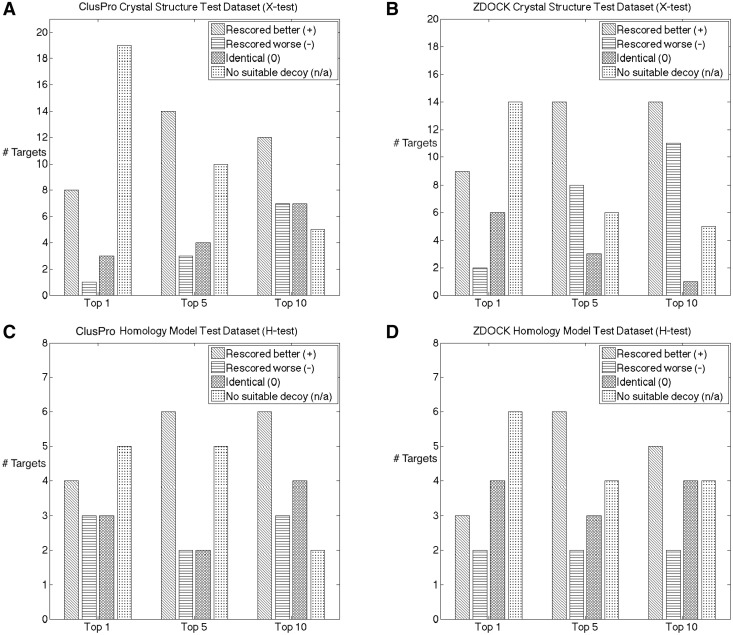
Success rates of rescoring compared with the raw decoy lists given by the docking algorithms. We show results for each docking program (ZDOCK or ClusPro) on each test set (X-test or H-test) for top one, five and ten results. The leftmost bars are the number of times our global docking pipeline improved results. Bars that are second to left are the corresponding number of cases when including epitope information made the results worse. Bars that are second to right are the number of times including the epitope information did not change the raw result. The rightmost bars are the number of cases for which both procedures reported no close-to-native decoys. See Supplementary Section 6 for the per-complex information. (**A**) Success rate of ClusPro on dataset X-test. (**B**) Success rate of ZDOCK on dataset X-test. (**C**) Success rate of ClusPro on dataset H-test. (**D**) Success rate of ZDOCK on dataset H-test

In Figure 2, we show how our methodology improves the standard docking procedures, which only make use of the paratope information. For instance, suppose that for a target in the top five poses as ordered by ClusPro, there are three close-to-native decoys, and in the corresponding top five results rescored using our pipeline, we obtain four close-to-native decoys. In such a case, our global docking pipeline produced an improvement by enriching the top results with more close-to-native decoys. If the number of close-to-native decoys is zero in both lists, we state that there were no suitable decoys.

As shown in Figure 2 on average, including epitope information improves over the raw results obtained by either ZDOCK or ClusPro. The improvement is particularly dramatic when considering the top scoring pose in the crystal structure dataset. Our rescoring brings eight close-to-native structures to the top position for ClusPro and nine for ZDOCK. This means that in almost a third of the cases, the top result is a close-to-native pose if rescored using our method.

The performance of the top prediction is not as pronounced in the homology model dataset, as our pipeline improves only slightly more cases than it deteriorates. The most pronounced increase in performance here is for the top five predictions, where we improve on 6 of the 15 cases for both ZDOCK and ClusPro. This suggests that using our method on a dataset closely resembling realistic input for virtual screening improves on the standard algorithms.

## 4 CONCLUSIONS

In this manuscript, we have demonstrated that our antibody-specific epitope prediction method is able to improve the global docking of antibodies and antigens.

The method EpiPred, when structures of the antibody and the antigen are given, annotates the likely epitope regions specific to the supplied antibody. In that respect, EpiPred differs from other methods such as DiscoTope or PEPITO, which annotate general immunogenic/epitope-like regions on the antigen, without any antibody information required on input (Kringelum *et al.*, 2012; Sela-Culang *et al.*, 2013). In particular, in comparison with DiscoTope 2.0, we demonstrate that including the antibody information appears to be advantageous for the prediction of antibody-specific epitopes.

We demonstrate that the top epitope predictions obtained using our method have a considerably higher average recall (44% recall at 14% precision) than that expected at random (23% recall at 14% precision) on a non-redundant set of crystal structures. We further demonstrate that EpiPred can receive homology models on input without a negative effect on performance. Thus, it appears that EpiPred requires only the sequence of an antibody and the structure of the antigen to produce meaningful results.

We have used the epitope predictions from EpiPred to rerank the outputs of two fast rigid-body docking algorithms and find that rescoring the decoys in this manner significantly enriches the number of close-to-native poses among the top one, five and ten results. This result holds for targets where the antibody is a homology model and the antigen is in the unbound structure. We have also tested our global docking pipeline on a blind test case supplied by UCB Pharma where once again including EpiPred predictions improved global docking results (see Supplementary Section 8).

In conclusion, our global pipeline increases the confidence that the close-to-native decoy will be among the top five poses. This is already a significant reduction of the potential set of possibilities experimentalists need to deal with when deciding on how to adjust the antibody sequence against the antigen. A researcher might choose to infer information by examining these or they could further refine the results using more time-consuming flexible docking procedures.

## Supplementary Material

Supplementary Data
