# Supplementary material for: Improving B-cell epitope prediction and its application to global antibody-antigen docking
Source: Bioinformatics. 2014 Apr 21;30(16):2288–94. doi: 10.1093/bioinformatics/btu190 (PMC4207425; doi:10.1093/bioinformatics/btu190)
Supplement: Supplementary Data [file supp_30_16_2288__index.html]

Improving B-cell epitope prediction and its application to global antibody-antigen docking — Improving B-cell epitope prediction and its application to global antibody-antigen docking — Improving B-cell epitope prediction and its application to global antibody-antigen docking — Supplementary Data 

# Improving B-cell epitope prediction and its application to global antibody-antigen docking

## Supplementary Data

files

**Files in this Data Supplement:**

- Supplementary Data - pdf file
