# Supplementary material for: Improving B-cell epitope prediction and its application to global antibody-antigen docking
Source: Bioinformatics. 2014 Apr 21;30(16):2288–94. doi: 10.1093/bioinformatics/btu190 (PMC4207425; doi:10.1093/bioinformatics/btu190)
Supplement: Supplementary Data [file supp_btu190_SUP.pdf]

Contents

1 Data **2**

1.1 SAbDab-nr 2

1.2 X-test 7

1.3 H-test 8

2 Sampling the patches on the antigen surface **9**

3 Calculating the precision score **11**

4 Inter-molecular distances **12**

5 Evaluating the performance of directionality of predictions. **14**

6 Supplementary tables **15**

7 Difference between the performance of EpiPred homology model and crystal structure datasets **15**

8 Evaluating the performance of the EpiPred and the global docking pipeline on a blind test case. **15**

1 Data

The datasets used in this study are presented in the sections below.

1.1 SAbDab-nr

The PDBs used in this study, SAbDab-nr: Table 1.

Table 1. Summary of the crystal structures used in this study, constituting SAbDab-nr.

PDB	Heavy chain	Light chain	Antigen Chain(s)	Antigen name
3d85	B	A	C	interleukin-23 subunit p19
1p2c	B	A	C	lysozyme c
3t3p	E	F	C	integrin alpha-iib
3zkm	C	D	B	beta-secretase 2
3gjf	H	L	A	hla class i histocompatibility antigen, a-2alpha chain
2uzi	H	L	R	gtpase hras
4i3s	H	L	G	outer domain of hiv-1 gp120 (ker2018 od4.2.2)
2vxs	J	N	D	interleukin-17a
1nsn	H	L	S	staphylococcal nuclease
3sqo	H	L	A	proprotein convertase subtilisin/kexin type 9
2jel	H	L	P	histidine-containing protein
1e6j	H	L	P	capsid protein p24
3lzf	H	L	A	hemagglutinin, ha1 subunit
3o2d	H	L	A	t-cell surface glycoprotein cd4
3eoa	H	L	I	integrin alpha-l
1nmb	H	L	N	n9 neuraminidase
3so3	C	B	A	suppressor of tumorigenicity 14 protein
3rkd	H	L	A	capsid protein
3lh2	I	M	T	4e10 1vi7a s0 002 n (t88)
3ngb	H	L	G	envelope glycoprotein gp160
4hwb	H	L	A	interleukin-13 receptor subunit alpha-1
3uc0	I	M	B	envelope protein
4hj0	P	Q	A	gastric inhibitory polypeptide receptor
3r1g	H	L	B	beta-secretase 1
3b9k	D	C	F	t-cell surface glycoprotein cd8 beta chain
4dkf	H	L	A	interleukin-34
1fj1	B	A	F	outer surface protein a
4g6m	H	L	A	interleukin-1 beta
3ma9	H	L	A	transmembrane glycoprotein
2xqy	G	L	A	envelope glycoprotein h

3hb3	C	D	B	cytochrome c oxidase subunit 2
1rjl	B	A	C	outer surface protein b
3i50	H	L	E	envelope glycoprotein
1n8z	B	A	C	receptor protein-tyrosine kinase erbb-2
1eo8	H	L	A	hemagglutinin (ha1 chain)
2xwt	A	B	C	thyrotropin receptor
3rvv	D	C	A	peptidase 1
4ag4	H	L	A	epithelial discoidin domain-containing receptor 1
4dtg	H	L	K	tissue factor pathway inhibitor
4ene	E	F	B	h(+)/cl(-) exchange transporter clca
4ffy	H	L	A	envelope glycoprotein
1ahw	E	D	F	tissue factor
2j88	H	L	A	hyaluronoglucosaminidase
1fsk	C	B	A	major pollen allergen bet v 1-a
3qwo	H	L	P	motavizumab epitope scaffold
1lk3	H	L	A	interleukin-10
3raj	H	L	A	adp-ribosyl cyclase 1
2ih3	A	B	C	voltage-gated potassium channel
2xqb	H	L	A	interleukin 15
3q1s	H	L	I	interleukin-22
1egj	H	L	A	cytokine receptor common beta chain precursor
1pkq	G	F	J	myelin oligodendrocyte glyco-protein
4ers	H	L	A	glucagon receptor
3sdy	H	L	B	hemagglutinin ha2 chain
2qqn	H	L	A	neuropilin-1
3lev	H	L	A	rna polymerase sigma factor
2j6e	H	L	A	ig gamma-1 chain c region
2h9g	B	A	R	tumor necrosis factor receptor superfamily member 10bprecur-sor
4hcr	M	N	B	mucosal addressin cell adhesion molecule 1
1tqb	B	C	A	prion protein
3hmx	H	L	A	interleukin-12 subunit beta
3p0y	H	L	A	epidermal growth factor receptor
2adf	H	L	A	von willebrand factor
3mxw	H	L	A	sonic hedgehog protein
1xiw	D	C	A	t-cell surface glycoprotein cd3 epsilon chain
3v6o	C	E	A	leptin receptor
2r0l	H	L	A	hepatocyte growth factor activa-tor

1h0d	B	A	C	angiogenin
3nfp	H	L	I	interleukin-2 receptor subunit alpha
4dn4	H	L	M	c-c motif chemokine 2
4d9q	E	D	B	factor d
2zch	H	L	P	prostate-specific antigen
3tt1	H	L	B	leucine transporter leut
2fd6	H	L	U	urokinase plasminogen activator surface receptor
4leo	A	B	C	receptor tyrosine-protein kinase erbb-3
4f3f	B	A	C	mesothelin
3mj9	H	L	A	junctional adhesion molecule-like
3kr3	H	L	D	insulin-like growth factor ii
3u9p	K	M	C	neutrophil gelatinase-associated lipocalin
3q3g	H	F	I	integrin alpha-m
2ypv	H	L	A	lipoprotein
1jrh	H	L	I	interferon-gamma receptor alpha chain
4hkx	A	B	E	hemagglutinin ha1
2qqk	H	L	A	neuropilin-2
3ru8	H	L	X	epitope scaffold 2bodx43
1iqd	B	A	C	human factor viii
4dw2	H	L	U	urokinase-type plasminogen activator
3cx5	J	K	E	cytochrome b-c1 complex subunit rieske,mitochondrial
2q8b	H	L	A	apical membrane antigen 1
4aei	I	M	B	alpha-mammal toxin aah2
3sob	H	L	B	low-density lipoprotein receptor-related protein 6
2yc1	D	E	F	beta-mammal toxin cn2
1v7m	H	L	V	thrombopoietin
1bgx	H	L	T	taq dna polymerase
3pnw	H	G	I	tudor domain-containing protein 3
4jr9	H	L	A	nitrite extrusion protein 1
2arj	B	A	R	t-cell surface glycoprotein cd8 alpha chain
3bgf	B	C	A	spike protein s1
1oaz	J	N	B	thioredoxin 1
4k2u	I	M	B	erythrocyte binding antigen 175
2r29	H	L	A	envelope protein e
4i9w	E	D	A	potassium channel subfamily k member 4
1kb5	H	L	A	kb5-c20 t-cell antigen receptor

3grw	H	L	A	fibroblast growth factor receptor 3
4f2m	C	D	F	spike protein
3ab0	B	C	A	bcla protein
4dqo	H	L	C	1fd6-v1v2 scaffold zm109 hiv-1 strain
1ors	B	A	C	potassium channel
1mhp	H	L	A	integrin alpha 1, (residues 169-360)
1fns	H	L	A	von willebrand factor
3vg9	C	B	A	adenosine receptor a2a
4etq	H	L	C	imv membrane protein
3liz	H	L	A	aspartic protease bla g 2
4i77	H	L	Z	interleukin-13
3s37	H	L	X	vascular endothelial growth factor receptor 2
3nh7	J	N	C	bone morphogenetic protein receptor type-1a
4am0	H	L	R	envelope protein,
3hi1	B	A	J	glycoprotein 120
2hmi	D	C	B	hisubunit of v-1 reverse transcriptase
4ffv	D	C	B	dipeptidyl peptidase 4
4hf5	H	L	A	hemagglutinin ha1
3ld8	C	B	A	bifunctional arginine demethylase and lysyl-hydroxylasejmjd6
4ht1	H	L	T	tumor necrosis factor ligand superfamily member 12
3o0r	H	L	B	nitric oxide reductase subunit b
3skj	H	L	E	ephrin type-a receptor 2
3dvg	B	A	Y	ubiquitin
1wej	H	L	F	cytochrome c
2r56	I	M	B	beta-lactoglobulin
1tzh	B	A	W	vascular endothelial growth factor a
4jpk	H	L	A	germline-targeting hiv-1 gp120 engineered outer domain,eod-gt6
3pgf	H	L	A	maltose-binding periplasmic protein
1yjd	H	L	C	t-cell-specific surface glycoprotein cd28
3vi3	H	L	D	integrin beta-1
2oz4	H	L	A	intercellular adhesion molecule 1
3ks0	H	L	B	cytochrome b2, mitochondrial
4g3y	H	L	C	tumor necrosis factor
3l95	H	L	Y	neurogenic locus notch homolog protein 1

4kuc	F	E	I	ricin
4fqj	H	L	A	hemagglutinin
1nfd	F	E	B	n15 alpha-beta t-cell receptor
4f37	H	L	A	colicin-e7 immunity protein
2vxq	H	L	A	pollen allergen phl p 2
1ob1	B	A	C	major merozoite surface protein
4jqi	H	L	A	beta-arrestin-1
2aep	H	L	A	neuraminidase
2vxt	H	L	I	interleukin-18
2nyy	D	C	A	botulinum neurotoxin type a
3gi9	H	L	C	uncharacterized protein mj0609

1.2 X-test

The data used in dataset X-test is presented in Table 2.

Table 2. Summary of the data constituting dataset X-test.

PDB	Heavy chain	Light chain	Antigen Chain(s)	Antigen name
1p2c	B	A	C	lysozyme c
3t3p	E	F	C	integrin alpha-iib
3zkm	C	D	B	beta-secretase 2
3gjf	H	L	A	hla class i histocompatibility antigen, a-2alpha chain
3o2d	H	L	A	t-cell surface glycoprotein cd4
4hj0	P	Q	A	gastric inhibitory polypeptide receptor
3r1g	H	L	B	beta-secretase 1
3ma9	H	L	A	transmembrane glycoprotein
3i50	H	L	E	envelope glycoprotein
1n8z	B	A	C	receptor protein-tyrosine kinase erbb-2
3rvv	D	C	A	peptidase 1
4ene	E	F	B	h(+)/cl(-) exchange transporter clca
3raj	H	L	A	adp-ribosyl cyclase 1
2ih3	A	B	C	voltage-gated potassium channel
3q1s	H	L	I	interleukin-22
3u9p	K	M	C	neutrophil gelatinase-associated lipocalin
1v7m	H	L	V	thrombopoietin
4jr9	H	L	A	nitrite extrusion protein 1
3ab0	B	C	A	bcla protein
1fns	H	L	A	von willebrand factor
3liz	H	L	A	aspartic protease bla g 2
4i77	H	L	Z	interleukin-13
4am0	H	L	R	envelope protein,
4ht1	H	L	T	tumor necrosis factor ligand superfamily member 12
3o0r	H	L	B	nitric oxide reductase subunit b
1tzh	B	A	W	vascular endothelial growth factor a
3pgf	H	L	A	maltose-binding periplasmic protein
4g3y	H	L	C	tumor necrosis factor
1nfd	F	E	B	n15 alpha-beta t-cell receptor
2vxt	H	L	I	interleukin-18

Bound complex PDB	Bound Ab chain (H,L)	Bound Ag chain	Unbound Ab PDB	Unbound Ab chain (H,L)	Unbound Ag PDB	Unbound Ag chain	Ag comment
1mlc	B A	E	1mlb	B A	1lza	A	Hen egg white lysozyme
1ahw	B A	C	1fgn	H L	1boy	A	Human tissue factor
1jps	H L	T	1jpt	H L	1tfh	A	Human tissue factor
1wej	H L	F	1qbl	H L	1hrc	A	Horse heart cytochrome C
1vfb	B A	C	1vfa	B A	8lyz	A	Hen egg white lysozyme
1bql	H L	Y	—-	- -	1dkj	A	Bobwhite quail lysozyme
1k4c	A B	C	—-	- -	1jvm	A	Potassium channel KcsA, in high concentration of K+
2jel	H L	P	—-	- -	1poh	A	Hpr, a phosphocarrier protein of the phosphoenolpyruvate:sugar phosphotransferase system of Escherichia coli
1jhl	H L	A	—-	- -	1ghl	A	Pheasant egg white lysozyme
1nca	H L	N	—-	- -	7nn9	A	Influenza A subtype N9 neuraminidase
2bdn	H L	A	—-	- -	—-	-	Small inducible cytokine A2
1ynt	B A	F	—-	- -	—-	-	Toxoplasma gondii surface antigen 1 (SAG1) p30
2aep	H L	A	—-	- -	—-	-	Neuraminidase (NA) of influenza virus A/Memphis/31/98 (H3N2)
2b2x	H L	A	—-	- -	—-	-	Integrin VLA1 RdeltaH I-domain
1ztx	H L	E	—-	- -	—-	-	Envelope protein of the West Nile virus

Table 3. Summary of the homology data in dataset H-test.

1.3 H-test

The summary of data constituting dataset H-test is given in Table 3.

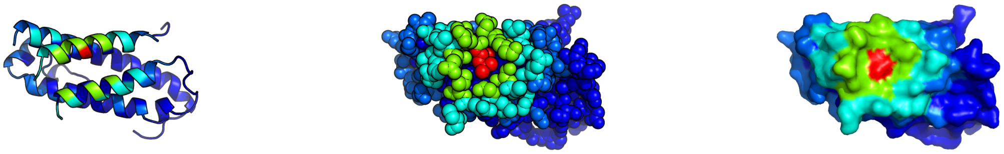

Figure 1. The visualization shows a single candidate patch on the surface, presented in three ways (cartoon, spheres and surface). This exemplifies the depth sampling used in this work: 4.5Å cutoff and depth of 3. The red residue is the central amino acid which initiates the patch (depth = 1). The green residues correspond to the neighborhood of the first residue (depth = 2). The teal residues are those within 4.5Å from the residues in green (depth = 3).

2 Sampling the patches on the antigen surface

Our method of sampling candidate surface patches consisted of extending each surface exposed residue on the antigen with the surface neighborhood. A surface neighborhood is created by successive additions of the surface residues within a certain cut-off distance to those already in the patch. Thus there are two parameters in this procedure: neighbor cutoff and the number of iterations of extending the neighborhood (depth). See Figure 1 for an example.
We have estimated the best parameter configuration for the patch sampling on our training set consisting of crystal structures (clearly excluding the entries in X-test). Using several values for these parameters, we have looked at average precision and recalls of the best, top five and top ten sampled patches given in Tables 4, 5 and 6 respectively.
We concluded that average precision in the region of 30% and recall in the region of 80% was achieved for the cut-off distance of 4.5Å and depth 3 which are used as standard parameters in the manuscript.

neighbor cutoff	depth	average precision (%)	average recall (%)
2.0	2	97	26
2.0	3	92	32
2.5	2	96	27
2.5	3	91	34
3.0	2	87	37
3.0	3	79	48
3.5	2	79	52
3.5	3	67	65
4.0	2	74	61
4.0	3	59	75
4.5	2	70	67
**4.5**	**3**	**52**	**80**

Table 4. Best sampled patches: the bast patch on SAbDab-nr minus X-test.

neighbor cutoff	depth	average precision (%)	average recall (%)
2.0	2	92	23
2.0	3	87	29
2.5	2	91	24
2.5	3	86	31
3.0	2	84	33
3.0	3	76	43
3.5	2	75	47
3.5	3	63	62
4.0	2	70	56
4.0	3	54	73
4.5	2	66	62
**4.5**	**3**	**48**	**79**

Table 5. Best sampled patches: out of 5 best on SAbDab-nr minus X-test.

neighbor cutoff	depth	average precision (%)	average recall (%)
2.0	2	86	20
2.0	3	83	25
2.5	2	85	21
2.5	3	81	27
3.0	2	79	29
3.0	3	72	39
3.5	2	71	42
3.5	3	59	59
4.0	2	64	52
4.0	3	50	71
4.5	2	60	58
**4.5**	**3**	**45**	**78**

Table 6. Best sampled patches: out of 10 best on SAbDab-nr minus X-test.

3 Calculating the precision score

We have performed local ZDOCK runs on each of the 118 targets. The constraints for the antibody are defined as the paratope residues extended by the surface residues within 5Å of it. The corresponding constraints for the antigen consist of the epitope, also extended by 5Å from it. Epitope and paratope are defined as sets of residues on the antigen and antibody respectively where for each residue in one set there exists at least one in the other whose inter-molecular distance to the first residue is less than 4.5Å . We have collected the top 200 decoys for each of the 118 targets, as ordered by ZDOCK, which resulted in a set of 23,600 decoys. Whenever an antibody-antigen residue pair (T_{ab}, T_{ag}) was observed within 4.5Å in any of the 23,600 decoys, we note down if it was a true positive (TP) or false positive (FP) with respect to the native structure (corresponding native contact is defined as within 4.5Å in the native structure for true positive). In this way, for each pair of residue types on the antibody and the antigen (T_{ab}, T_{ag}), we obtain the number of times ZDOCK matched them correctly, denoted as $TP(T_{ab}, T_{ag})$ and the corresponding number of incorrect matches given as $FP(T_{ab}, T_{ag})$. We use the number of true positives and false positives to define the precision score for a given antibody-antigen residue pair as given by 1:

$$Pr(T_{ab}, T_{ag}) = \frac{TP(T_{ab}, T_{ag})}{TP(T_{ab}, T_{ag}) + FP(T_{ab}, T_{ag})} \tag{1}$$

An analogous procedure was applied to train EpiPred for use with H-test, by removing those pdbs from SAbDab-nr that shared more than 90% sequence identity with the antigens or more than 99% sequence identity with antibodies in H-test.

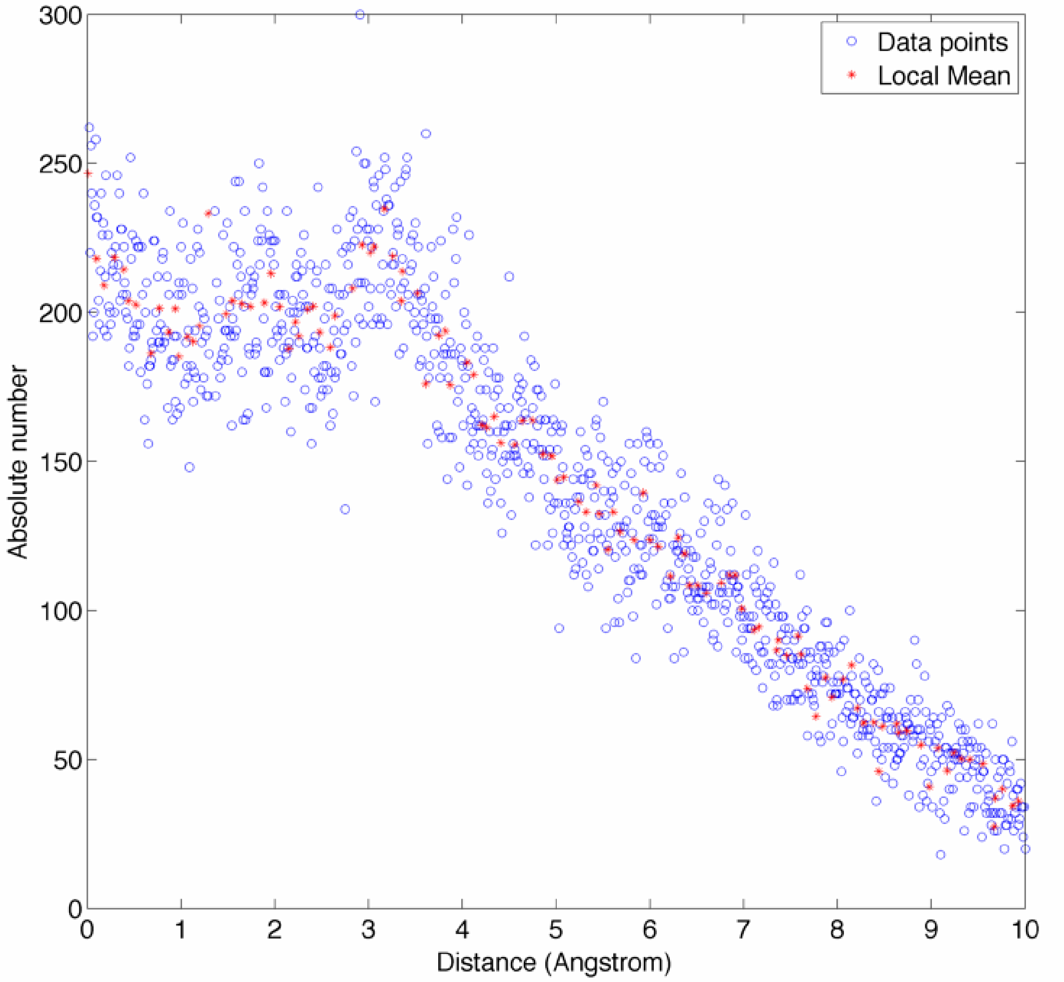

Figure 2. Histogram of the distribution of differences between intra-molecular distances between pairs of interacting residues. The blue points are the absolute numbers of times a given distance was observed whereas the red ones indicate a local mean of such points.

4 Inter-molecular distances

In order to motivate our choices of parameters for EpiPred, we have estimated the distribution of inter-molecular distances between pairs of interacting residues. Take node n_1 which stands for a contact between antibody residue r_{ab1} and antigen residue r_{ag1} and node n_2 with antibody residue r_{ab2} and antigen residue r_{ag2}. Define $dist(r_{ab1}, r_{ab2})$ as the intra-molecular distance between the two residues r_{ab1} and r_{ab2}. Define $dist(r_{ab1}, r_{ab2})$ as the intra-molecular distance between the two residues r_{ab1} and r_{ab2}. Figure 2 gives the distribution of intra-molecular distance differences $(|dist(r_{ab1}, r_{ab2}) - dist(r_{ag1}, r_{ag2})|)$ for each pair of interacting residues in dataset SAbDab-nr that were not in X-test.

It appears that majority of the differences fall in the region of 0 to 3Å after which point the numbers become lower. For this reason we have tested EpiPred on the structures in SAbDab-nr that were not in X-test using several intra-molecular difference cutoffs: 0.1Å, 0.5Å, 1.0Å, 1.5Å, 2.0Å, 2.5Å and 3.0Å. EpiPred achieved similar results for cut-offs of 1Åand higher with poorer results (close to random) for cut-offs of 0.1Å and 0.5Å. Since the lowest cut-off (1Å) which produced satisfying results was least computationally expensive, it is used as the default cut-off in EpiPred.

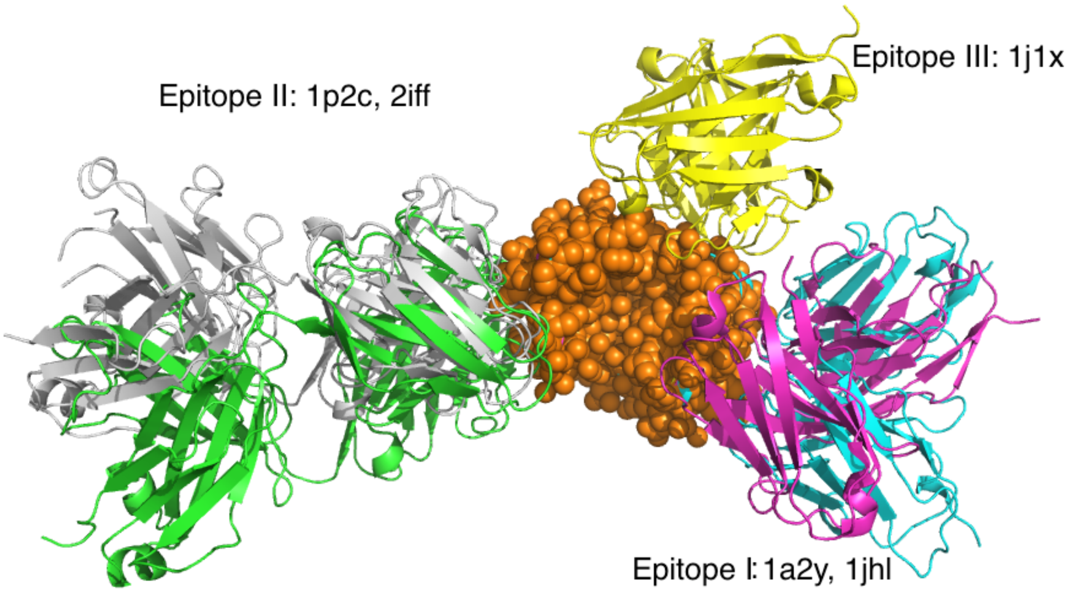

Figure 3. The three epitopes used for the directionality study and their representatives.

5 Evaluating the performance of directionality of predictions.

There are eight binding modes of antibodies to lysozyme found in the PDB, (see Figure 3), five of them are full antibodies and three are camelid. The five non-camelid binding modes which bind to three distinct epitopes were used for the analysis presented in the manuscript.

6 Supplementary tables

The detailed docking results tables are given in 7 for X-test and 8 for H-test. Tables contrasting the results with lengths of H3 are given in as Tables 9 and 10.

7 Difference between the performance of EpiPred homology model and crystal structure datasets

In order to evaluate how different performance of EpiPred is on X-test and H-test, we have compared their sample average precisions and recalls. In order to achieve the estimates of the average means and precision, we have sampled precision and recalls values from both datasets. For instance, one sample for the average precision and recall of X-test would consist of 30 precisions and recalls sampled at random with replacement form the precision-recall pair values available in X-test. For each sample of 30 precision-recall pairs we have recorded the average precision and recall from those 30 pairs. Similar procedure was applied to H-test.
In total we have sampled 10^7 averages from X-test and H-test. We have fitted a line through the precision-recall pairs for the averages of X-test and H-test (see Figure 4). The lines plotted in this way for X-test and H-test cannot be called statistically significantly different when their slope and intercept are compared.

8 Evaluating the performance of the EpiPred and the global docking pipeline on a blind test case.

Our collaborators from UCB Pharma provided us with a blind test case to evaluate our epitope prediction and global docking pipelines. We were given a sequence of the antibody and the crystal structure of the antigen it forms a complex with. The antigen structure was an asymmetric homo-dimer. For both epitope prediction and docking, we needed a structure of the antibody. This structure was modeled using PIGS ([1]). We have not used RosettaAntibody due to its unavailability at the time ([2]). We have not modeled H3 using FREAD ([3]) as the programs library did not have appropriate fragments for the this particular instance of the loop.
We have predicted the top three epitope patches using standard parameters of EpiPred used throughout this manuscript. The top epitope prediction is incorrect placing the candidate epitope on the wrong end of the asymmetric homo-dimer structure of the antigen. Nevertheless, the second one overlaps greatly with the actual epitope (see Figure 5).
We have performed docking of the antibody homology model to the antigen structure using ClusPro ([4]). There was only one decoy out of 28 returned with I_{rmsd} at 10.645Å which was low enough to be tentatively classified as close to native . All the other decoys had considerably higher I_{rmsd} values. This decoy was at seventh position as ordered by ClusPro, but was brought to the third position using our re-scoring pipeline. This top decoy superimposed on the native complex is shown in Figure 5.

			Epitope Prediction						ZDOCK									ClusPro								
			Top 1st		Top 2nd		Top 3rd		T1			T5			T10			T1			T5			T10		
PDB	Ag size	Best epitope	Precision	Recall	Precision	Recall	Precision	Recall	Raw	Rescored	Comparison	Raw	Rescored	Comparison	Raw	Rescored	Comparison	Raw	Rescored	Comparison	Raw	Rescored	Comparison	Raw	Rescored	Comparison
4hj0	92	1	32	90	0	0	-	-	1	1	(0)	5	3	(-)	9	5	(-)	0	1	(+)	0	2	(+)	2	3	(+)
1tzh	94	2	1	6	72	81	-	-	0	1	(+)	2	4	(+)	4	7	(+)	0	0	n/a	3	3	(0)	6	5	(-)
4am0	96	-	13	70	0	0	-	-	0	1	(+)	3	2	(-)	5	4	(-)	1	1	(0)	3	2	(-)	7	5	(-)
2ih3	97	1	16	64	0	0	-	-	1	1	(0)	5	5	(0)	9	10	(+)	0	1	(+)	0	4	(+)	1	5	(+)
4i77	97	1	23	55	31	50	-	-	1	1	(0)	3	4	(+)	4	7	(+)	0	1	(+)	1	2	(+)	5	5	(0)
3q1s	113	1	19	81	-	-	-	-	1	1	(0)	1	3	(+)	3	5	(+)	0	0	n/a	1	2	(+)	3	4	(+)
1p2c	129	2	0	0	34	87	-	-	0	1	(+)	2	4	(+)	3	6	(+)	1	1	(0)	2	3	(+)	3	3	(0)
4ht1	131	2	5	14	50	95	-	-	0	1	(+)	1	2	(+)	1	2	(+)	0	0	n/a	0	1	(+)	1	1	(0)
3ab0	136	1	33	73	41	73	0	0	0	1	(+)	1	2	(+)	1	4	(+)	0	1	(+)	0	2	(+)	3	5	(+)
1v7m	145	1	26	77	5	11	6	11	0	1	(+)	1	2	(+)	2	3	(+)	1	0	(-)	2	1	(-)	3	2	(-)
4g3y	148	2	3	8	51	82	0	0	0	0	n/a	0	1	(+)	0	1	(+)	0	0	n/a	0	0	n/a	0	0	n/a
2vxt	156	2	4	9	45	95	7	14	1	1	(0)	2	5	(+)	6	10	(+)	0	1	(+)	0	1	(+)	0	1	(+)
3u9p	169	1	31	100	0	0	0	0	0	1	(+)	2	2	(0)	5	4	(-)	0	0	n/a	1	1	(0)	3	1	(-)
3o2d	178	1	32	64	12	20	10	16	0	0	n/a	1	0	(-)	3	1	(-)	0	0	n/a	1	0	(-)	2	1	(-)
1fns	196	-	0	0	0	0	8	27	0	0	n/a	2	1	(-)	4	1	(-)	0	0	n/a	0	0	n/a	3	1	(-)
3ma9	197	-	0	0	12	27	11	22	1	1	(0)	4	5	(+)	9	8	(-)	0	1	(+)	1	2	(+)	3	4	(+)
3rvv	223	1	25	93	2	6	7	20	0	0	n/a	0	0	n/a	0	0	n/a	0	0	n/a	1	1	(0)	2	2	(0)
3raj	230	2	0	0	19	75	15	50	0	0	n/a	0	2	(+)	0	2	(+)	0	0	n/a	0	0	n/a	0	0	n/a
1nfd	239	-	7	23	0	0	0	0	0	0	n/a	0	0	n/a	1	0	(-)	0	0	n/a	0	0	n/a	2	2	(0)
3i50	273	3	0	0	0	0	25	100	1	0	(-)	4	0	(-)	7	3	(-)	0	0	n/a	0	0	n/a	0	0	n/a
3gjf	276	1	15	66	0	0	12	27	0	0	n/a	0	0	n/a	0	0	n/a	0	0	n/a	0	1	(+)	2	2	(0)
3liz	329	1	26	68	0	0	34	78	0	0	n/a	0	1	(+)	0	1	(+)	0	1	(+)	1	3	(+)	2	3	(+)
3pgf	358	-	2	4	8	18	11	22	0	0	n/a	4	2	(-)	8	4	(-)	0	0	n/a	0	0	n/a	0	1	(+)
3zkm	375	1	32	88	0	0	0	0	0	1	(+)	1	4	(+)	6	7	(+)	0	1	(+)	1	2	(+)	2	3	(+)
3r1g	381	1	37	100	0	0	0	0	0	1	(+)	1	5	(+)	5	8	(+)	0	0	n/a	2	3	(+)	3	4	(+)
4jr9	409	1	19	85	0	0	0	0	0	0	n/a	2	2	(0)	4	4	(0)	1	1	(0)	2	4	(+)	6	8	(+)
4ene	442	-	0	0	0	0	0	0	0	0	n/a	0	0	n/a	0	0	n/a	0	0	n/a	0	0	n/a	1	0	(-)
3o0r	449	-	8	70	0	0	0	0	0	0	n/a	0	0	n/a	0	0	n/a	0	0	n/a	0	0	n/a	1	1	(0)
3t3p	453	-	0	0	2	5	0	0	0	0	n/a	1	0	(-)	1	0	(-)	0	0	n/a	1	1	(0)	1	2	(+)
1n8z	581	-	0	0	0	0	0	0	1	0	(-)	2	0	(-)	4	1	(-)	0	0	n/a	0	0	n/a	0	0	n/a

Table 7. Table summarizing the results of epitope prediction and global docking on the X-test dataset. We present the top three epitope predictions returned by EpiPred. For smaller antigens, it was impossible to return more than one or two top epitopes as a result of overlap cut-off set at 30%. The best epitope field refers to the first occurrence of a suitable epitope prediction among the top three returned. The global docking results are annotated with indications of whether the re-scored list of decoys was better or not with the following meanings: **(+)** re-scoring improved the result, **(-)** re-scoring made the result worse, **(0)** re-scoring did not improve the result and there exist close to native decoys, **(n/a)** no suitable decoys were available.

			Epitope Prediction						ZDOCK									ClusPro								
			Top 1st		Top 2nd		Top 3rd		T1			T5			T10			T1			T5			T10		
PDB	Ag size	Best epitope	Precision	Recall	Precision	Recall	Precision	Recall	Raw	Rescored	Comparison	Raw	Rescored	Comparison	Raw	Rescored	Comparison	Raw	Rescored	Comparison	Raw	Rescored	Comparison	Raw	Rescored	Comparison
2bdn	68	1	21	52	29	41	-	-	1	0	(-)	1	1	(0)	1	1	(0)	0	0	n/a	1	1	(0)	3	4	(+)
2jel	82	1	25	92	-	-	-	-	0	0	n/a	1	3	(+)	2	4	(+)	1	1	(0)	3	4	(+)	7	9	(+)
1k4c	97	3	12	37	0	0	44	75	0	1	(+)	2	4	(+)	5	6	(+)	0	1	(+)	0	1	(+)	0	1	(+)
1ztx	101	2	4	11	23	58	25	52	0	1	(+)	2	4	(+)	3	7	(+)	0	1	(+)	1	2	(+)	5	6	(+)
1wej	101	-	10	66	-	-	-	-	0	0	n/a	1	1	(0)	1	2	(+)	1	1	(0)	1	3	(+)	3	3	(0)
1jhl	126	2	11	40	29	93	0	0	1	1	(0)	5	4	(-)	9	7	(-)	0	0	n/a	1	1	(0)	3	3	(0)
1mlc	126	1	29	82	20	52	0	0	1	0	(-)	1	1	(0)	2	2	(0)	0	0	n/a	1	1	(0)	3	4	(+)
1bql	126	2	10	29	27	70	0	0	1	1	(0)	1	3	(+)	1	5	(+)	0	1	(+)	1	4	(+)	2	5	(+)
1vfb	126	-	18	38	5	9	24	42	1	1	(0)	2	3	(+)	7	7	(0)	1	0	(-)	3	2	(-)	6	4	(-)
2b2x	188	3	0	0	21	47	25	52	0	1	(+)	2	1	(-)	6	2	(-)	1	0	(-)	2	0	(-)	2	1	(-)
1jps	200	1	22	59	20	45	0	0	0	0	n/a	0	0	n/a	0	0	n/a	1	0	(-)	3	1	(-)	5	4	(-)
1ahw	200	1	36	94	10	26	28	68	0	0	n/a	0	0	n/a	0	0	n/a	1	1	(0)	2	2	(0)	5	5	(0)
1ynt	252	3	0	0	6	13	29	52	1	1	(0)	1	2	(+)	4	4	(0)	0	1	(+)	1	2	(+)	2	2	(0)
2aep	358	-	8	18	11	22	0	0	0	0	n/a	0	0	n/a	0	0	n/a	0	0	n/a	0	0	n/a	0	0	n/a
1nca	388	1	42	84	38	68	0	0	0	0	n/a	0	0	n/a	0	0	n/a	0	0	n/a	0	0	n/a	0	0	n/a

Table 8. Table summarizing the results of epitope prediction and global docking on the H-test dataset. We present the top three epitope predictions returned by EpiPred. For smaller antigens, it was impossible to return more than one or two top epitopes as a result of overlap cut-off set at 30%. The best epitope field refers to the first occurrence of a suitable epitope prediction among the top three returned. The global docking results are annotated with indications of whether the re-scored list of decoys was better or not with the following meanings: **(+)** re-scoring improved the result, **(-)** re-scoring made the result worse, **(0)** re-scoring did not improve the result and there exist close to native decoys, **(n/a)** no suitable decoys were available.

		Epitope Prediction								
		EpiPred			DiscoTope 2.0			Random		
PDB	Ag size	Precision (%)	Recall (%)	MCC	Precision (%)	Recall (%)	MCC	Precision (%)	Recall (%)	Chothia CDR-H3 length
4hj0	92	**32**	**90**	0.27	0	0	0.0	29	50	10
1tzh	94	1	6	0.04	**73**	**87**	0.72	12	25	13
4am0	96	**13**	**70**	0.09	33	20	0.19	14	60	8
2ih3	97	**16**	**64**	0.08	0	0	0.0	15	27	9
4i77	97	**23**	**55**	0.0	0	0	0.0	21	31	10
3q1s	113	**19**	**81**	0.15	0	0	0.0	20	37	17
1p2c	129	0	0	0.0	**100**	**5**	0.0	39	32	7
4ht1	131	**5**	**14**	0.05	0	0	0.0	28	44	11
3ab0	136	**33**	**73**	0.34	0	0	0.0	33	52	8
1v7m	145	**26**	**77**	0.29	0	0	0.0	9	16	5
4g3y	148	3	8	0.04	**100**	**17**	0.33	11	25	9
2vxt	156	4	9	0.04	**47**	**36**	0.3	14	23	4
3u9p	169	**31**	**100**	0.47	6	5	0.0	8	15	11
3o2d	178	**32**	**64**	0.28	0	0	0.0	9	16	13
1fns	196	0	0	0.0	**100**	**7**	0.0	33	11	14
3ma9	197	0	0	0.0	0	0	0.0	21	33	12
3rvv	223	**25**	**93**	0.39	15	17	0.07	6	15	12
3raj	230	0	0	0.0	0	0	0.0	24	21	11
1nfd	239	7	23	0.04	**92**	**70**	0.75	10	15	9
3i50	273	0	0	0.0	0	0	0.0	1	6	10
3gjf	276	**15**	**66**	0.2	5	11	0.05	6	15	10
3liz	329	**26**	**68**	0.34	0	0	0.0	10	15	10
3pgf	358	**2**	**4**	0.0	0	0	0.0	13	18	11
3zkm	375	**32**	**88**	0.46	0	0	0.0	11	15	8
3r1g	381	**37**	**100**	0.57	0	0	0.0	7	9	10
4jr9	409	19	85	0.34	**46**	**50**	0.46	4	11	9
4ene	442	0	0	0.0	0	0	0.0	1	5	12
3o0r	449	**8**	**70**	0.19	0	0	0.0	2	14	15
3t3p	453	0	0	0.0	**25**	**4**	0.0	4	6	10
1n8z	581	0	0	0.0	0	0	0.0	6	5	11

Table 9. Table summarizing the results of epitope prediction on the X-test set. We present the top EpiPred prediction and the corresponding results for DiscoTope 2.0 using a score threshold of -3.7. The values in bold indicate the best prediction result. Precision and recall were computed by the following formulas: $precision = TP/(TP + FP)$, $recall = TP/(TP + FN)$ where TP stands for true positives, FP for false positives and FN for false negatives. In each case we also give the Matthews Correlation Coefficient (MCC). As control, the corresponding result using randomized score is give for each target. This Table corresponds to Table 1 in the manuscript. Lengths of CDR-H3 are given for each structure so as to provide a contrast between the prediction results and the relative difficulty of modeling of this loop.

PDB	Ag size	Precision/ Recall (%)	MCC	Chothia CDR-H3 length	PDB	Ag size	Precision/ Recall (%)	MCC	Chothia CDR-H3 length
2bdn (B)	68	21/52	0.1	8	2jel (U)	82	25/92	0.2	9
1k4c (U)	97	12/37	0.1	9	1ztx (B)	101	4/11	0	10
1wej (U)	101	10/66	0.16	8	1jhl (U)	126	11/40	0.13	9
1mlc (U)	126	29/82	0.33	7	1bql (U)	126	10/29	0.06	7
1vfb (U)	126	18/38	0.11	8	2b2x (B)	188	0/0	0	10
1jps (U)	200	22/59	0.21	8	1ahw (U)	200	36/94	0.49	8
1ynt (B)	252	0/0	0	9	2aep (B)	358	8/18	0.02	9
1nca (U)	388	42/84	0.55	11	-	-	-	-	0

Table 10. Table summarizing the results of epitope prediction on the H-test set. Precision and recall were computed by the following formulas: $precision = TP/(TP + FP)$, $recall = TP/(TP + FN)$ where TP stands for true positives, FP for false positives and FN for false negatives. In each case we also give the Matthews Correlation Coefficient (MCC). Lengths of CDR-H3 are given for each structure so as to provide a contrast between the prediction results and the relative difficulty of modeling of this loop.

The results from this single blind test case are in favor of our claim that including epitope predictions improves the results of global antibody-antigen docking.

References

1. Marcatili P, Rosi A, Tramontano A (2008) Pigs: automatic prediction of antibody structures. Bioinformatics 24: 1953–1954.

2. Sivasubramanian A, Sircar A, Chaudhury S, Gray JJ (2009) Toward high-resolution homology modeling of antibody fv regions and application to antibody-antigen docking. Proteins 74: 497–514.

3. Choi Y, Deane CM (2010) Fread revisited: Accurate loop structure prediction using a database search algorithm. Proteins 78: 1431–40.

4. Brenke R, Hall D, Chuang G, Comeau S, Bohnuud T, et al. (2012) Application of asymmetric statistical potentials to antibody–protein docking. Bioinformatics 28: 2608–2614.

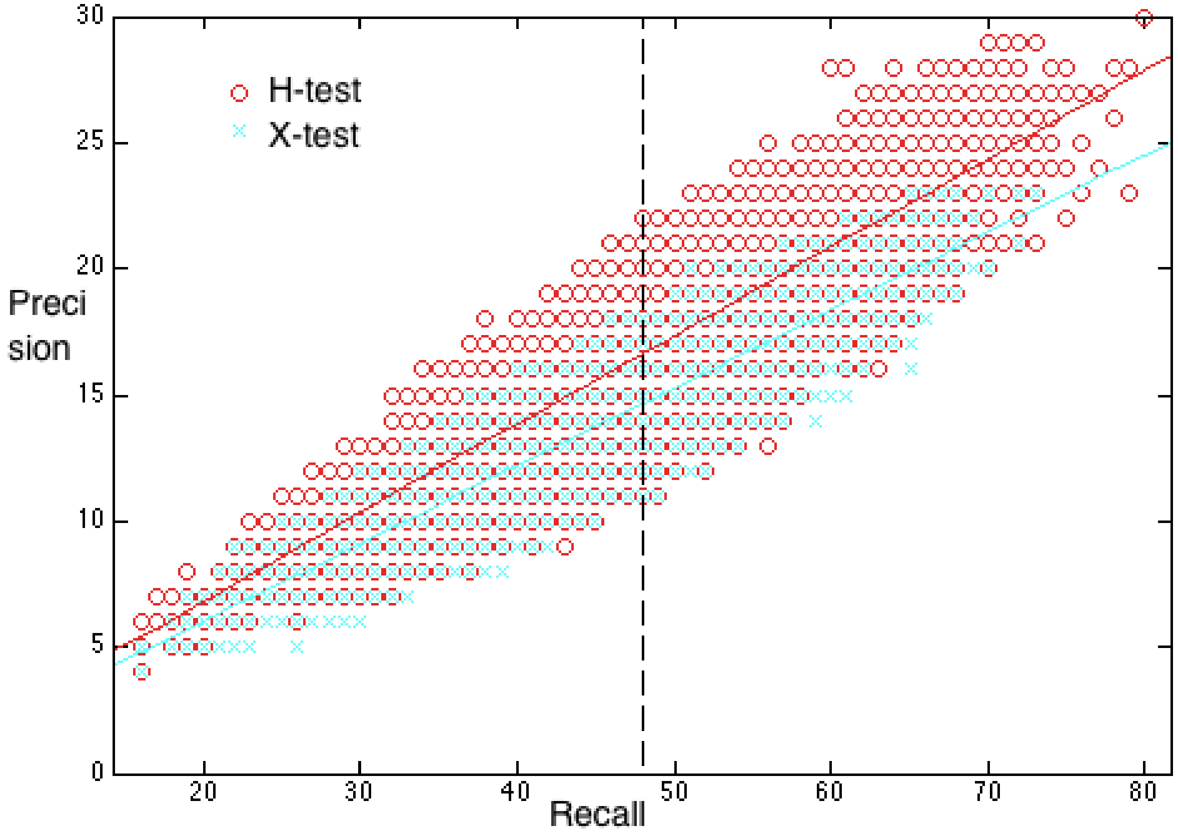

Figure 4. The plot of samples of mean precision and recall values for X-test and H-test with best-fit lines indicated.

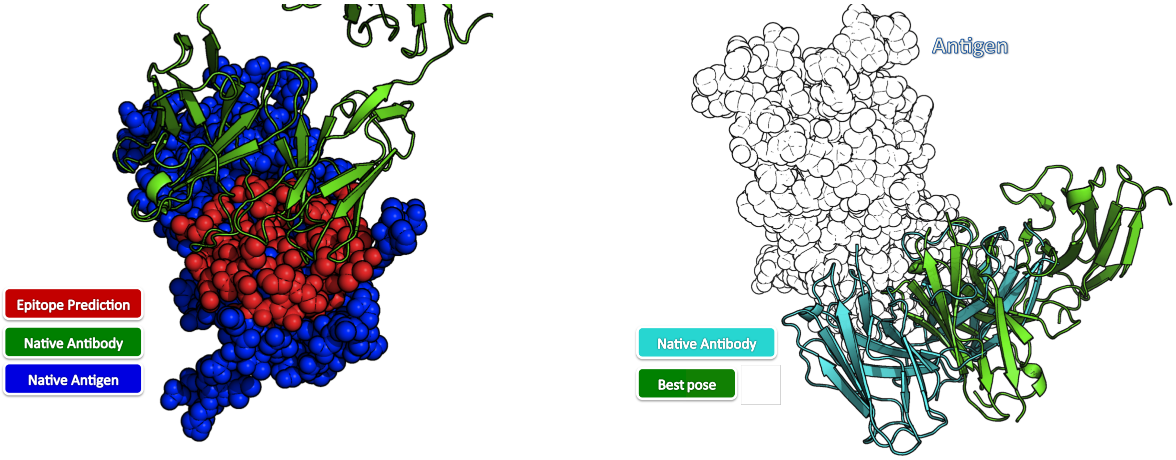

Figure 5. **Left:** The top second epitope prediction on the blind test-case is shown in red. Note that it covers the region where the actual epitope is as indicated by the native contacting antibody (in green). This epitope prediction achieved 52% precision and 69% recall. **Right:** The best decoy returned by ClusPro (green) contrasted with the native position of the antibody (teal). Notice that the antibody is rotated correctly and the discrepancy is only due to lateral translation.
